# Child-mother relationships and childhood dietary patterns in the Iberian Peninsula uncovered by Bayesian isotopic approaches

**DOI:** 10.1038/s41598-025-97967-4

**Published:** 2025-04-13

**Authors:** Alice Toso, Silvia Casimiro, Charlotte Oxborough, Simona Schifano, Maite I. García-Collado, Francisca Alves Cardoso, Joaquina Soares, Maria João Valente, Raquel Santos, Vanessa Filipe, Maria José da Silva Gonçalves, Nuno Neto, Paulo Rebelo, Rodrigo Banha da Silva, Anabela Novais de Castro Filipe, Michelle Alexander

**Affiliations:** 1 Bonn Center for ArchaeoSciences, Institut für Archäologie und Kulturanthropologie, Rheinische Friedrich-Wilhelms-Universität, Bonn, Germany; 2 IEM - Institute for Medieval Studies, School of Social Sciences and Humanities, Nova University of Lisbon, Portugal (NOVA FCSH) / LABOH CRIA – Center for Research in Anthropology (NOVA FCSH)/ Atalaia Plural - Archaeology, Heritage & Territory, Lisbon, Portugal; 3 Department of Archaeology. BioArCh, University of York, Environment Building, 2nd floor. Wentworth Way, YO10 5DD Heslington, UK; 4 Department of Geography, Prehistory and Archaeology. Research group in Medieval Archaeology, Heritage and Cultural Landscapes (IT442-22)., University of the Basque Country (UPV/EHU), Micaela Portilla Research Centre. 1 Justo Vélez de Elorriaga street., 01006 Vitoria-Gasteiz, Spain; 5 LABOH CRIA – Center for Research in Anthropology, School of Social Sciences and Humanities (NOVA FCSH), Nova University of Lisbon, Portugal/ In2PAST - Associated Laboratory for Research and Innovation in Heritage, Arts, Sustainability and Territory, Lisbon, Portugal; 6 Faculdade de Letras, UNIARQ - Centro de Arqueologia da Universidade de Lisboa, Universidade de Lisboa, Lisbon, Portugal; 7 Universidade do Algarve / CEAACP — Centro de Estudos de Arqueologia, Artes e Ciências do Património, Faro, Portugal; 8 Neoépica, Mem Martins, Portugal; 9 School of Social Sciences and Humanities (NOVA FCSH), Cota 80-86, IAP – Instituto de Arqueologia e Paleociências, Nova University of Lisbon, Lisbon, Portugal; 10 Museu de Arqueologia de Silves, Silves, Portugal; 11 CHAM – Center for Humanities, School of Social Sciences and Humanities, Nova University of Lisbon (CHAM - NOVA FCSH), Lisbon, Portugal; 12 Lisbon Archaeology Centre, (DMC/DPC/CAL), Lisbon City Council, Portugal; 13 Atalaia Plural, Lisbon, Portugal

**Keywords:** Bayesian modelling, Childhood diet, Portugal, Weaning, Breastfeeding, Biogeochemistry, Biochemistry, Environmental social sciences

## Abstract

This study examines trends in infant diet, breastfeeding and weaning in Portugal through time in Roman, Medieval Muslim and Christian skeletal assemblages (1st to the 15th century CE). New stable carbon (δ^13^C) and nitrogen (δ^15^N) measurements were collected from 143 non-adults and 46 adults that are analysed alongside comparative published datasets from contemporaneous Iberian populations. A statistical package was used to model bone collagen nitrogen isotope data of individuals, quantitatively estimating weaning onset and completion across diverse historical sites. Nutritional intake from infancy to adolescence was reconstructed via Bayesian modelling supported by the OsteoBioR platform using incremental dentine-collagen isotope ratio analysis in six adult individuals. Childhood diets in historical Portugal showed a prolonged weaning time while weaning food included varying degrees of high trophic level protein during both the Roman and Medieval periods. The Bayesian statistical approach offers a comprehensive perspective on child-rearing practices through the lens of diet, including breastfeeding, weaning and nutritional intake during childhood in historical Portugal. The results highlight the variability and complexity of childhood diets over time and between different locations. Overall, the study informs debates about child nutrition practices globally while also offering unique insights into infant nutrition in Iberia over nearly 1500 years.

**Supplementary Information:**

The online version contains supplementary material available at 10.1038/s41598-025-97967-4.

## Introduction

In recent decades, there has been a growing interest in the archaeology and anthropology of childhood, with recent research challenging traditional narratives that marginalize the role of children role in past societies. This research has emphasized the importance of recognizing children’s social and economic agency within their communities, which undermines earlier assumptions about child dependency and passivity as immutable constructs^1,2^.

The examination of infant diets allows for the reconstruction of various aspects of daily life in archaeological populations. This information aids in understanding local dietary traditions from a socio-cultural perspective and how childcare may differ across socio-economic contexts^3^. Additionally, it provides important insights into population demography, nutrition patterns, and disease prevalence among mothers and offspring^4,5^. Breastfeeding habits are closely intertwined with societal culture as well as family traditions; they also serve as a means for self-regulation by fertile women to plan their families’ social status and wellbeing^6–8^. Deciding when to wean an infant off breast milk can be complex since it affects an infant’s ability to cope with nutritional or physiological stress^9^, while mothers have to balance the investment in the current offspring with the future reproductive prospects^10^. Introducing complementary foods is a critical stage of a child’s development, but may pose risks such as pathogens invading the immature immune system of a developing child^11^.

Breastfeeding was highly valued for child development and health in Roman, Muslim and Christian Medieval societies, and was performed by mothers and wet-nurses, especially among high status families or when no other option was available^12–14^. Medical treatises describe best practices for breastfeeding and weaning often recommending breastfeeding until the child is two years of age^15,16^. Our previous isotopic study confirmed that Medieval (7th -11th century CE) non-adults from Lisbon, similar to other archaeological populations and, in keeping with the medical recommendations, were fully weaned from two years of age at the earliest and in some cases not until three years of age^17^. The results also suggested that a special weaning diet with a higher intake of C_4_ plants might have been consumed by high-status infants in Lisbon during this period, although we could find no mention of this practice in historical or medical literature^18,19^.

This study sets out to investigate two key aspects of early life dietary practices in southern Iberia. First, it aims to reconstruct the predominant dietary sources consumed by non-adult individuals across different temporal phases, with a particular focus on southern Portugal. Second, it explores whether isotopic signatures can elucidate patterns of infant feeding, notably the duration of breastfeeding and the nature of weaning foods, and how these may have varied in response to shifting historical and socio-cultural conditions.

### Bioarchaeological research into childhood diet

Stable isotope analysis, specifically that of carbon (δ^13^C) and nitrogen (δ^15^N), has become a popular method for investigating breastfeeding and weaning behaviours in archaeological populations. The fundamental concept underlying this approach is that during the period of exclusive breastfeeding, an infant consumes maternal tissues (breastmilk) resulting in an increase of δ^15^N values by approximately one trophic level (2–3‰) compared to the mother^20,21^. This phenomenon was notably observed through studies analysing hair and nail samples from modern populations^21,22^. However, chemical changes that occur within infant tissues throughout the weaning process are influenced by various factors such as exclusive breastfeeding practices or consumption of supplementary foods and drinking water^23^. The progressive replacement of breastmilk protein with protein from other food sources, which are typically at a lower trophic level than breastmilk, can lead to a decrease in infant δ^15^N values that aligns with the adult levels in a population, as a result of adults and weaned infants consuming similar diets^20^. Although carbon isotope profiles have been hypothesised to be a more robust indicator of weaning age in archaeological populations, the impact on δ^13^C values is expected to be less significant, resulting in only about a 1‰ trophic level shift^21,24^. A child is deemed fully weaned once breastfeeding has ceased. After this stage, the child’s tissues isotopic composition will primarily be derived from their diet, metabolism as well as drinking water intake. These values may resemble those found among the adult population but can also vary according to regional cultural practices and traditions which influence access to diverse food options^17^.

In bioarchaeological studies, two primary methods for examining breastfeeding and weaning patterns have traditionally been applied: cross-sectional analysis and incremental analysis. Cross-sectional analysis involves the collection of bulk bone samples from non-adult individuals of varying ages to compare with adult values, providing a general overview of population-wide weaning periods and overall childhood diet^3,24,25^. Incremental dentine-collagen isotope ratio analysis (IDIA) offers a more individual-level insight into feeding practices by sampling non-remodelling tissues like dentine in increments, to produce high-resolution isotopic values at different formation times^26–29^. Recent applications of incremental analysis have yielded significant contributions toward understanding how physiological stressors, such as disease or malnutrition, affect isotopic values^28,30,31^.

The constraints associated with cross-sectional analysis of bone collagen, from a skeletal population, with the aim of reconstructing breastfeeding and weaning practices, have been thoroughly discussed in scholarly circles. The primary concerns center around the challenge of accurately interpreting isotopic data, given that particular values may be explicable through a variety of equally plausible circumstances^32,33^. Additional challenges include inaccuracies in aging technique, which can lead to over- or underestimation of an individual’s true age – particularly when pathology is known to impact bone/teeth development; non-diet-related physiological processes that may affect isotopic shifts between mothers and offspring; and poorly understood bone turnover rates^29,34,35, 36^. It is also worth noting that stable isotope values obtained from a single bone sample are unlikely to provide a complete representation of an individual’s dietary inputs across their lifespan. This is because such samples incorporate tissue deposited during pregnancy, as well as during breastfeeding and weaning phases - with variations likely depending on differences in skeletal turnover rates throughout the body. As a result, it can be difficult to record actual dietary changes that happened in a short time span^37^.

Moreover, when estimating weaning time, it is assumed that the female population represents the females that gave birth and that the diet of adult females, in a specific context, is homogenous. This often leads to generalizing nursing patterns for an entire sample or certain groups of individuals within a dataset, while disregarding individual variation and cultural practices influenced by social status, gender roles, or health conditions. Since identifying specific infant-mother pairs is rare in the archaeological record, non-adult values are compared with an overly homogeneous adult baseline, which may result in misinterpreting outliers with notably high or low values. Additionally, the non-adults under analysis represent non-survivors who might have had different metabolic and life histories than survivors and therefore caution must be exercised when analysing such data^24,29,38,39^. To mitigate some of these concerns, incremental sampling approaches can be employed to document life stages that extend before those directly preceding death. Nevertheless, despite these possible challenges, analysing cross-sectional isotopic data remains the most effective approach for comprehending average weaning times in historical populations when longitudinal isotopic data from dentine Sects^29,40^ are unavailable.

It is worth stressing that while weaning and breastfeeding practices play a crucial role in evolutionary biology research and serve as pivotal developmental milestones for children, comprehensive cross-sectional studies encompassing all age groups of non-adults still hold significant value in investigating childrearing customs and ways of life throughout history. In this paper we opted to include all age groups beyond the period of weaning to provide a more comprehensive overview of the overall diet before adulthood, assumed in this paper to be the age at which humans reach skeletal maturity (18–20 years old). To circumvent some of the limitations explained above, we have also opted for including a small set of dentine serial analysis of individuals that have survived into adulthood from the burial site in Beja and to use Bayesian modelling to enhance the methodological approach, as described below.

### Childhood diet in historical times

Breastfeeding and weaning practices were not often discussed in historic texts. Fortunately, there is a handful of textual sources which offer some insights into these processes in the past. For example, religious texts include references to breastfeeding. Books in the Bible written between the fifth and second centuries BCE (2 Maccabees 7:27, 2 Chronicles 31:16) describe a three-year period of nursing. Later sources from the first millennium CE such as Quran (II:233,31 − 14) and Babylonian Talmud (Ketubboth60a V5) suggest that this period lasted two years^41,42^. Avicenna (10th /11th c. CE) advocated that the introduction of solid food must coincide with the eruption of a child’s first incisors, and weaning ought to be completed when they reach two years old^43^. Breastfeeding was viewed as crucial for the health and growth of children by Christian and Muslim Medieval societies, and wet-nurses were employed when mothers could not nurse, or in particularly affluent households. Scholarly works from both traditions advocate breastfeeding until at least two years of age, based on historical and medical evidence^12–16^.

In terms of later childhood through to adulthood, Greek and Roman authors wrote about recommended diets based on the individual’s age and gender, following the prevailing medical theories of the time, although the degree of adoption of these recommendation by the general population is largely unknown^44^. For instance, Galen and Hippocrates suggested that men should consume “wet and cold” marine resources to maintain balance since they are classified as “hot and dry,” whereas women who are inherently deemed “wet and cold” ought to avoid these marine products and instead focus more on cereals and terrestrial proteins for a healthy diet, especially after puberty^44^. Dietary recommendations may have been associated with perceived life stages rendering the use of a life course perspective in dietary reconstruction particular poignant. Infants were supposed to receive dry and cool foods to balance their wet and hot humours, while young people should have consumed cool and wet foods for the same purpose. During the Roman Empire the male life cycle was divided into six distinct stages depending on the physical and mental development of the individual, as expressed by the work of Isidore of Seville (560–630 CE)^45^. The female’s life course was divided into only two stages and referring to adult life: *virgos* (virginal but physically mature woman), and *puerpera* (pregnant women). Additional life stages have been mentioned (i.e. wife and mother), but are loosely characterised^46^. Evidence from bioarchaeological research challenges the oversimplified understanding of dietary practices in ancient populations presented by historical texts. The findings highlight variations among different groups, such as disparities between adult males and females^47,48^, as well as differences between non-adults and adults within the same population^49,50^. For instance, at Isola Sacra near Rome, there were notable differences in diet between males and females from childhood, and non-adult individuals had more constrained diets compared to adults^51–53^. There are limited reviews on dietary practices during the Roman Empire and Late Antique period^54,55^, with most case studies focused on Roman Britain or Italy, revealing distinct local variation that includes also non-adult diets, showing different feeding patterns related to age and economic instability^49,50,56–58^. Much of our understanding of food practices in the Western Roman Empire come from texts, table and cookware, as well as plant and animal remains from archaeological excavations^59–64^. A recent review of isotopic data for the *Hispania* province^65^ highlighted the complexity of dietary practices in the Iberian Peninsula during this time period. This study also emphasizes the need to further investigate and expand knowledge about dietary practices in the Roman and Late Antique periods in order to gain a more detailed picture^66^. Similar calls for more comprehensive dietary studies for Iberian historical populations have been made previously by researchers studying this region’s history, whose results for the Roman, Late Antique and Early Medieval periods, demonstrated continuity with previous and subsequent historical periods. To address this call for action, our study takes a diachronic approach to examine the dietary practices of non-adult individuals in Lisbon and its surrounding regions. This includes the incorporation of new stable isotope data on bulk bone and dentine collagen to a previously published corpus of isotopic analysis for the same region. By comparing isotopic data across age groups, we can gain insights into the dietary practices and potential variations among different segments of the population during periods of political and economic change, as well as document the long-term childrearing practices patterns and nutritional status of non-adults in this region.

## Materials and methods

**Fig. 1:**
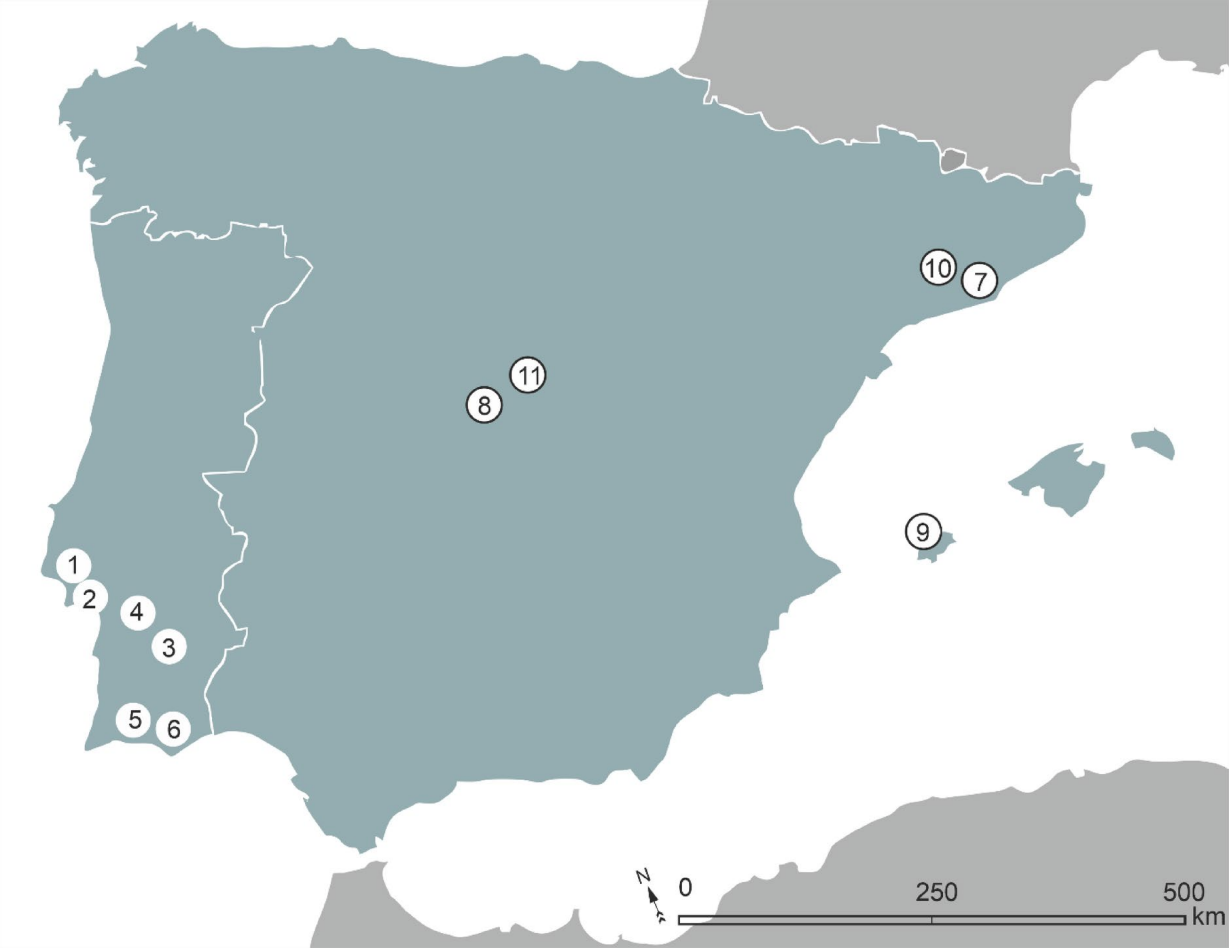
Map – 1 Lisbon; 2 Setúbal; 3 Beja; 4 Torrão; 5 Silves; 6 Loulé; 7 Barcelona; 8 Boadilla; 9 Ibiza; 10 Terassa, 11 Gozquez. The closed circles indicate previously published sites. Bibliographic references for these can be found in Table 1.

Detailed site and samples descriptions are provided for all the newly generated data in supplementary information (SI). Our analysis also incorporates isotopic data from several locations that have previously been the subject of isotopic publications including Lisbon, Beja and Silves^17,67^ therefore, only brief site descriptions are provided. Other available published contemporaneous Roman and Late Roman non-adult isotopic data, from a range of sites of the Iberian Peninsula and Ibiza, have been used in the discussion as comparative datasets and cited in Table 1, while a full list of the isotopic values can be found in the SI. The geographical distribution of all datasets is shown in Fig. 1.

**Table 1:** List of the analysed sites and individuals in the present site, as well as published comparative data sets with indication of chronology, number of individuals per age group and bibliographic reference.

Location	Period	Non-adults < 10 years	Non-adults > 10 years	Adults	Reference
Lisbon	Roman (1st to 6th)	39	1	24	This study
Lisbon	Early Med (8–12)	15	4	15	^17^
Setúbal	Early Med (8–12)	5	0	4	This study
Beja	Early Med (8–12)	16	6	21	This study and^67^
Loulé	Early Med (8–12)	6	0	7	This study
Torrão	Early Med (8–12)	4	2	11	This study
Silves	Early Med (8–12)	25	6	7	This study and^67^
Lisbon	Late Med (13–15)	5	2	20	This study and^67^
Beja	Late Med (13–15)	6	3	7	This study and^67^
Silves	Late Med (13–15)	14	4	22	This study and^67^
Barcelona	Roman (2nd-4th)	8	0	15	^47^
Terrassa	Roman (5th-8th)	8	0	28	^68^
Ibiza	Roman (3th-7th)	17	8	35	^69^
Boadilla	Early Medieval (5th-8th)	8	4	49	^70^
Gozquez	Early Medieval (6th-8th)	8	1	6	This study and García-Collado, 2016
Total		184	36	271	

### Sample selection

Age-at-death was, for most cases, recovered from the anthropological reports of the sites under analysis, while co-authors S.C. and F.A.C. have undertaken the archaeological and bioarchaeological study of the Roman sites. For all included populations age ranges were assigned using standards based on tooth formation and eruption^71^, bone fusion^72^ and long bone length^73–75^. Age categories were defined as follows: fetus (under 28 gestation weeks), perinate (between 28 and 42 gestation weeks), infant (0–1 years), child (1–7), child (8–14), adolescent (14–18)^76,77^. For statistical purposes, we have defined additional groupings for all non-adults : 0–1 year; between 1.1 and 3 years; between 3.1 and 5 years; between 5.1 and 10 years; adult (from 18 years of age). This grouping has been chosen because it identifies different developmental stages of children, that either trigger different dietary needs, or have been linked in historical sources to distinct life stages. During the first year of life, it was strongly encouraged that children would receive breastmilk exclusively, while solid food could be slowly introduced towards the end of the first year^78^. Longer weaning has however been confirmed by several studies with a complete cessation of breastfeeding at an age of around three years, with some cases suggesting weaning was completed around the age of four, especially in the Late Antique period^79^. In order to be able to identify prolonged weaning practices, we have therefore added two more groups up to the age of three and five respectively. Between the age of five and ten, children ended the early childhood (*infantia*) and entered a functional childhood (*pueritia*) during which small tasks could have already been performed, especially within the household^80^. For both males and females, in the Roman period, childhood could end as early as ten years old, as this was generally the age at which child slaves were regarded as useful enough to be traded as such^81^, while female children could legally marry as early as 12 years old^53^. For these reasons, the last group considered as part of childhood was up to ten years old.

Bone from a hundred and forty-one non-adults was sampled (Table 1). Well-preserved ribs or rib fragments were preferred, however, especially in particularly young individuals, other bones have been sampled where ribs were not available or poorly preserved. The non-adult samples were complemented by forty-six newly sampled adult female individuals that acted as a baseline for the breastfeeding calculations, in addition to the already published adult data from the same sites^17,67^. A full list can be found in SI. From these, 100 mg of bone shards were sampled and mechanically cleaned with a scalpel blade, removing the outer 1–2 mm of bone.

A small subset of six adult teeth have also been analysed, through incremental dentine analysis, to provide a control sample of individuals that survived into adulthood^29^. Since this work is looking into not only breastfeeding and weaning but also later childhood dietary biographies, a range of teeth were selected that formed over a range of ages in adulthood^82^. M1 were chosen to explore the first years of life. The temporal framework for M2 and PM pertains to a subsequent phase during the initial stage of childhood, nevertheless, an inquiry into early childhood becomes pertinent as breastfeeding is observed to persist at these locations. If the mother has exclusively breastfed her child and not altered her own diet prior to weaning, then there will be a marked increase in δ^15^N values during this first increment. Subsequently, as solid foods are introduced and breastfeeding decreases, isotopic values tend to decline over time^28^.

### Collagen extraction and stable isotope analysis

For bone samples a modified Longin^83^ method, which included an added ultrafiltration step^84^, was utilized to extract collagen. Bones were demineralized at 4ºC in 0.6 M HCl, followed by rinsing to neutrality and gelatinization in HCl at pH 3 and a temperature of 80 °C. The resulting gelatinized fraction underwent ultrafiltration using Amicon 30 kDa filters and the retentates removed, frozen ( ~ − 20 °C) and lyophilized for analysis. Dentine samples were obtained following^28^. Teeth were cleaned, dried and sectioned along the mesio-distal plane using a Buehler IsoMet1000 slow-speed water saw with a diamond blade. Enamel was removed with a Dremel drill, and the remaining sample was rinsed, dried and demineralised using 0.6 M HCl. Teeth were then sectioned with horizontal cuts of 1 mm and each section was heated in a pH3 HCl solution at a temperature of 80 °C for 48 h. The gelatinised fractions were frozen, lyophilised and weighed to determine the percent yield. Using isotope ratio mass spectrometry (IRMS) with a Sercon 20–22 instrument at the BioArCh facilities within the University of York, bone and dentine collagen samples were analysed for isotopic values following standard practice guidelines as δ values in parts per mille, relative to internationally defined standards for carbon-^13^/^12^C (VPDB: Vienna Pee Dee Belemnite) and nitrogen-^15^/^14^N(AIR). Data was normalised using a two-point linear correction using repeated measurements of international standard reference materials as calibration standards within each analytical run (in-house fish gelatine: δ^13^C − 15.5 ± 0.1‰, δ^15^N 14.3 ± 0.2‰; cane sugar IA-R006: δ^13^C − 11.8 ± 0.1‰; caffeine IAEA 600: δ^13^C − 27.8 ± 0.1‰, δ^15^N 0.8 ± 0.1‰; ammonium sulphate IAEA N2: δ^15^N 20.4 ± 0.2‰). Sample uncertainty for individual samples are calculated separately for both δ^15^N and delta δ^13^C using the Kragten spreadsheet model^85^ as outlined in the Good Practice Guide for Isotope Ratio Mass Spectrometry^86^, https://www.forensic-isotopes.org/gpg.html) by combining uncertainties in the values of the reported error associated with the published true value of the international standards, the measured error (precision) values of the international standards in the run, the precision error from the measurement of the two sample replicates. These are expressed as one standard deviation. The maximum uncertainty for all samples across all runs was < 0.2‰ for both δ^13^C and δ^15^N. Collagen from all individuals in this study had acceptable C: N ratios, as well as %C and %N^87,88^.

Initial age estimation of dentine sections was completed by using the stage of dental development provided by the London Atlas^89^ and the sample’s anatomical location. Three age points were set following the London Atlas: at completion of crown, crown-root junction and completion of root. The number of dentine sections obtained for each segment were dived by the number of months for each of these three segments to achieve a more precise age estimation^82^. The incremental growth of dentine follows a cone-like pattern, thus horizontal or oblique cuts will results in some inevitable blending between age points^90^. For this reason, assigning a specific age of formation to each dentine section is often not as precise as would be desirable. However, dental development a highly reliable process, which is only impaired or slowed in cases of severe malnutrition^91^, whose traces have not been found on the skeletal remains of the sampled individuals. Furthermore, to minimise the impact of this sampling method on the isotopic estimation, the software OsteoBioR was specifically used to statistically account for the different contribution of age points to the same horizontal dentine section.

### Statistical analysis

Statistical analysis was performed in R (version 4.0.3; packages ggplot, WARN). Comparison of δ^13^C and δ^15^N values between samples were carried out using one-way ANOVA, Wilcoxon rank sum and Kruskal-Wallis tests (α = 0.05), after checking for normal distribution with Shapiro-Wilk test for normality (α = 0.05). The R package WARN was utilised to model data^37^ from non-adult individuals below the age of ten. When age was reported as an age range in the anthropological report, a mean age was estimated. The software OsteoBioR^56^ (available at https://isomemoapp.com) was employed to model the dentine increments from six individuals from the Early and Late Medieval site of Beja.

## Results

### Non-adult and adult diet

**Fig. 2:**
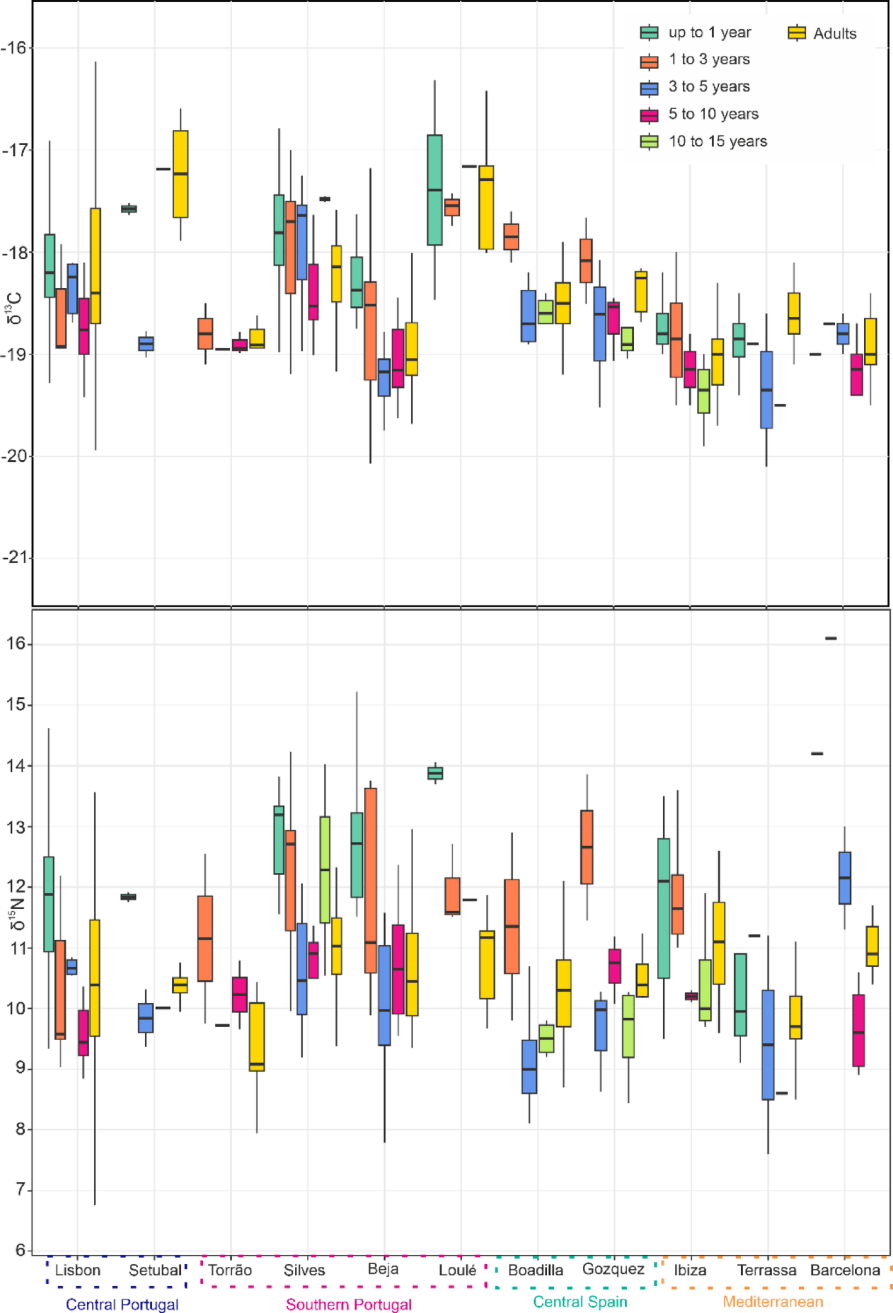
Box plot comparison of δ^13^C and δ^15^N values of non-adults from different age categories and adults (A) individuals from several sites (West to East) dating between the 1st and the 15th century CE. The dataset includes Lisbon (*n* = 120) (this study^17^, ;^67,92^, Setúbal (*n* = 9), Beja (*n* = 55) (this study and Toso et al. 2021), Torrão (*n* = 17), Silves (*n* = 78) (this study and Toso et al. 2021), Loulé (*n* = 13) (this study), Barcelona (*n* = 23)^47^, Terrassa (*n* = 36)^68^, Ibiza (*n* = 60)^69^, Boadilla (*n* = 63)^70^, Gozquez (*n* = 18)(this study and^93^.

The diets of both adult and non-adult individuals exhibit significant intra- and inter-variability across all time periods, as demonstrated by Fig. 2.

Adults δ^13^C and δ^15^N values range between − 21.0‰ and − 15.8‰ and 6.4‰ and 13.6‰, respectively, whereas those for non-adults up to one year of age fall within − 19.4‰ and − 16.3‰ for δ^13^C and 8.2‰ and 15.2‰ for δ^15^N. Individuals aged between one and three years display varying values in terms of both parameters with a range from − 20.1‰ and − 17‰ for δ^13^C and 9‰ and 16.1‰ for δ^15^N (Fig. 2). Non-adult individuals between three to five years show values of -20.1‰ and − 16.6‰ for δ^13^C and from 7.6‰ to 13.6‰ for δ^15^N, closer to the range of the adult population. Non-adult aged between five to ten years have a more terrestrial range of δ^13^C values between − 21.2‰ and − 17.2‰ and δ^15^N values that fall within 8.1‰ and 14‰. Values of individuals in late childhood and early adolescence (between 10 and 15 years of age) have an even smaller range of δ^13^C values between − 20‰ and − 18.4‰ and δ^15^N values between 9.2‰ and 12‰, respectively.

The consumption of^13^C-enriched foods was found to be higher among both adult and non-adult populations in Lisbon, Silves, Loulé and Setúbal compared to other groups, with the former two locations showing the most enriched values among all sites. Coastal locations suggest that marine protein played a significant role in the diet of these settlements. However, it is noteworthy that Setúbal’s adult population consumed a larger proportion of this type of protein compared to the non-adults aged three to five years old. The inland sites of Torrão and Beja, but also the sites of Ibiza, Terrassa and Barcelona were identified as having the lowest δ^13^C values for both adults and non-adults, and Terrassa exhibited highly terrestrial δ^13^C values especially among non-adults aged three to five years. Boadilla and Gozquez show non-adults aged between one and three years old to have higher values than the other age categories at these sites, however the difference is less than 1‰ and not statistically significant, suggesting some variation in carbon sources within terrestrial food sources. The distribution of δ^15^N values across various locations unveils intriguing trends. Notably, the highest values for individuals up to one year were observed in Silves, Beja, Loulé, followed by Lisbon and Ibiza. Non-adults between one and three years old exhibited higher δ^15^N values in Silves, Beja and Gozquez, followed by Torrão, Boadilla and Ibiza. The adult populations of the coastal sites of Setúbal and Loulé that showed the highest values for δ^13^C, do not show comparatively high values of δ^15^N suggesting that lower trophic level marine resources are most likely responsible for this distribution, with the exception of non-adults up to one year old from Loulé who maintain higher values of δ^15^N due to breastfeeding. In the population of Boadilla and Gozquez, non-adults between one and three years of age show the highest δ^15^N values, indicating that a prolongation of breastfeeding might have occurred at these sites, although the lack of individuals aged one and below hinders further comparison.

### Breastfeeding and weaning practices

**Fig. 3:**
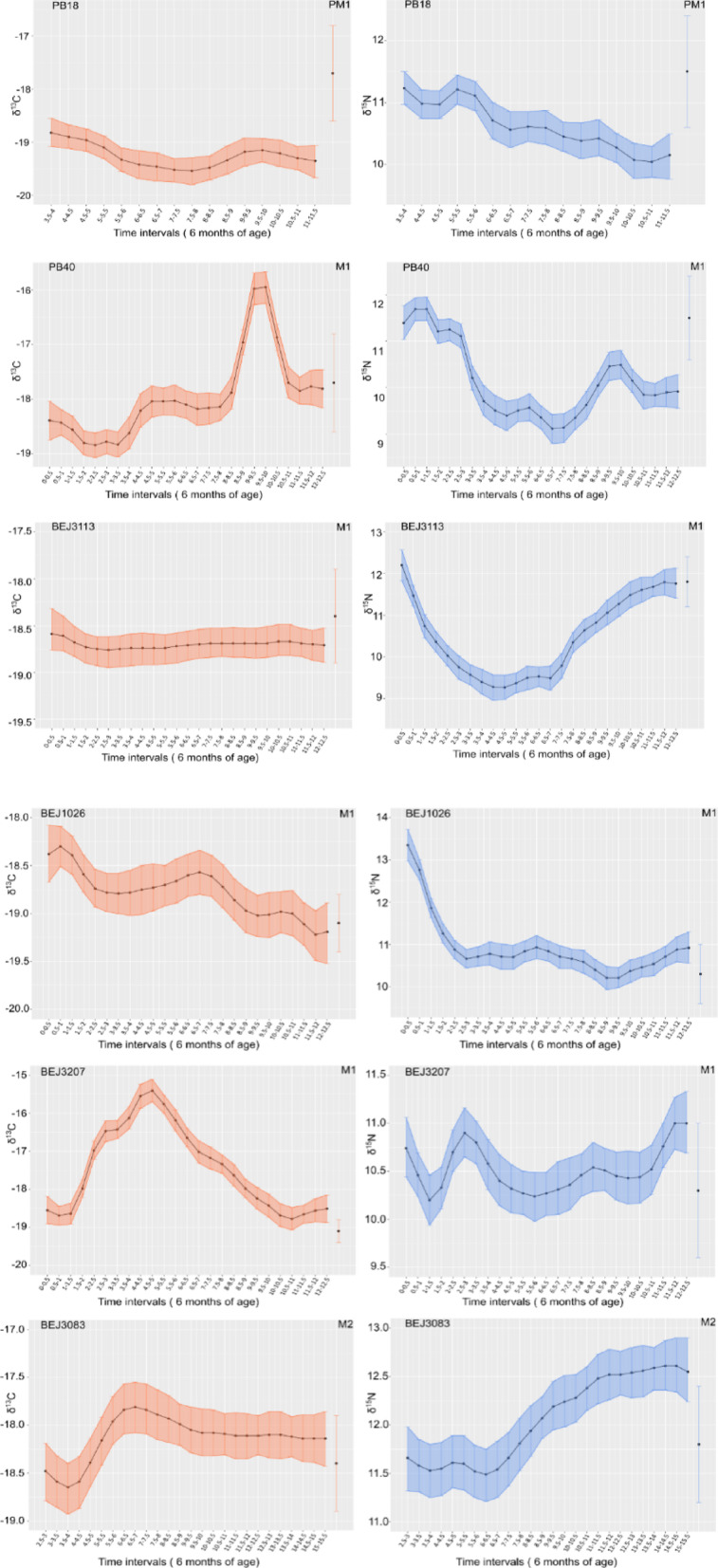
Bayesian temporal modelling of incremental dentine δ^13^C and δ^15^N profiles of one first premolar (PB18) and one first molar (PB40) from Lisbon (Poço do Borratém 11th -15th c.), and three first molars (BEJ3113, BEJ1026, BEJ3207) and one second molar (BEJ3083) from Beja (Escola Secundaria Diogo Gouveia 8th -15th c.).

**Fig. 4:**
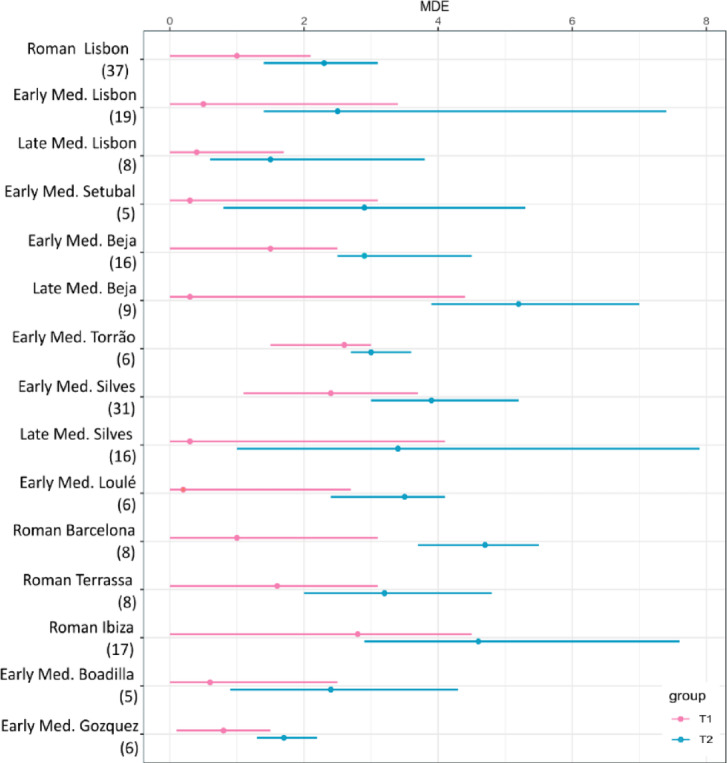
Ranges for the onset (t1) and completion of weaning (t2) including the maximum density estimates from all data sets analysed with the WARN R Package. The dataset includes Roman Lisbon (this study), Early Medieval Lisbon^17^, Late Medieval Lisbon^67,92^, Setúbal, Beja, Torrão, Silves, Loulé (this study), Barcelona^47^, Terrassa^68^, Ibiza^69^, Boadilla^70^, Gozquez (this study and^93^.

Great variability in breastfeeding and weaning timing was estimated across all sites (Figs. 3 and 4). For each of the data sets, individual isotopic values and results from the application of the WARN R package and OsteoBioR are presented in SI (SI Table 1). Breastfeeding timing (SI Fig. 1) was estimated with the R Package WARN and weaning curves were generated for each site by chronological period taking into consideration changes in baseline and adult diet. From this model, breastfeeding in the Lisbon assemblages seems to have been practiced until one and a half year of age in the Roman and Early Medieval period, while for the Late Medieval period exclusive breastfeeding ended before one year of age. The nearby Early Medieval population of Setúbal shows estimations consistent with breastfeeding up to two years of age, followed by the progressive decrease of nitrogen values indicating the onset of a supplementary weaning diet. Early Medieval populations in Beja and Torrão show a similar pattern to Setúbal, while Loulé and Silves show a more gradual decrease in nitrogen values, consistent with predominantly breastfeeding until three years of age. Equally prolonged breastfeeding was estimated for Late Medieval Beja, Silves, and the Roman sites in Barcelona, Terrassa and Ibiza. The Early Medieval sites of Boadilla and Gozquez show a pattern of exclusive breastfeeding up to one and a half year of age.

The analysis of our dataset revealed a diverse range of practices, which aligns with the findings from WARN. Notably, none of the individuals examined exhibited a typical breastfeeding pattern characterized by increasing δ^15^N values and stable or slightly higher δ^13^C values. Our estimation based on WARN indicated that while weaning was extended beyond what was commonly observed in medical texts at that time (approximately 2 years), it also began relatively early. The distribution of δ^13^C and δ^15^N values in the first tooth increments for all individuals suggests this trend holds true as well since their mean δ^15^N value closely mirrors that of the female population’s during infancy before decreasing consistently over time (PB40, BEJ3113, BEJ3207, BEJ1026). The δ^13^C values exhibit a comparable pattern and are already at birth within the range of the female population. If we abstain from taking the female mean into account, because of the problematic methodological association of using female mean as a ‘mother’ mean, we can observe that the lowest δ^15^N values indicating complete weaning, occur between 4 and 9 years of age. As for the post-weaning stage, most individuals display similar values as those in the female population by age 10. Notably, three individuals (PB40, BEJ3207 and BEJ3083) experienced a sudden increase in their δ^13^C levels around nine, four and six years respectively which suggests an intake of^13^C-enriched food. The PB40 profile also indicated higher levels of marine protein based on increased δ^15^N value; however this was not observed simultaneously in the other two cases thereby suggesting consumption of different food sources such as C_4_ plants. In addition, four out of the six individuals show a steep increase in δ^15^N values at around age seven that could be occurring before the pubertal growth spurt. This pattern has been observed elsewhere^56,94^, and studies on animal tissues have shown an inverse correlation between growth rate and^15^Nenrichment^30,95^. The WARN model was additionally able to provide a measure of the^15^N enrichment between the female adult mean and the non-adult mean of individuals up to three years of age to explore trophic level relationships in the different data sets. Although the dentine samples did not show much difference between these two parameters, the modelled MDE for^15^N enrichment was found to be the highest in Barcelona, Gozquez, Loulé and Torrão. Literature indicates that^15^N-enrichment varies in modern infant-mother pairs, ranging between 1.7‰ and 2.8‰ ^21^. Archaeological examples also show variable values, with a range of 0.5‰ to 4.4‰ ^96^; populations above these reported values may suggest the addition of higher trophic level food into the diet of non-adults at weaning onset. Quantitative analysis of the data using the R Package WARN at each site by chronological period suggests that the onset and completion of weaning follows significantly different patterns at all sites (Fig. 4). In Lisbon weaning started (t1) between 0 and 2.1 years and was completed (t2) between the ages of 1.4 and 3.1 during the Roman period. The onset of weaning seems to remain similar also during the Early (0-3.4 years) and Late (0-3.1 years) Medieval period, however the completion of weaning was not carried out until 5.3 and 4.5 years of age during the Early and Late Medieval period respectively. Similarly, extended weaning period can be observed for other Portuguese Early and Late Medieval sites such as Setúbal, Beja, Silves and Loulé. Among the Roman sites, Barcelona and Ibiza present the longest weaning practices for Eastern Iberia.

## Discussion

### Interpreting breastfeeding practices in Iberia

Breastfeeding is regarded as a means of maternal investment in infants, which engenders a conflict between the mother and child, as the former strives to optimize her current offspring’s support while securing future reproductive opportunities. This phenomenon has resulted in diverse patterns of breastfeeding and weaning across mammalian species, determined by ecological factors, social norms, life history traits, unique to each individual or species^10,97^. A comprehensive exploration into variation among individuals’ weaning timelines can offer valuable insights regarding various adaptive and reproductive strategies employed under varying environmental conditions, as well as evolving socio-political frameworks. In this regard it is particularly beneficial, whenever possible, to adopt a diachronic approach within the same geographical locations in order to explore the impact of diverse social and historical processes on a specific population through time. This was the case for Lisbon, where we could analyse sites from the Roman to the Late Medieval period. During the Roman and Medieval periods, breastfeeding was practiced until one and a half year, while in the Late Medieval period breastfeeding was ended at the mark of 12 months of age. This trend of an early termination of breastfeeding in Late Medieval Lisbon is also compatible with the dentine data of individual PB40 (Fig. 3), showing decreasing values of both isotopes at one and a half years of age, while individuals PB18, at three years of age, already showed values within the range of the adult population. We presume that these different feeding behaviours in Lisbon would have had an impact on the fertility of the population, and these practices may have led to breastfeeding mothers resuming their fertility sooner and devoting more time to economic activities. Studies on medieval women in Portugal, and especially their family roles and legal status, have found that women enjoyed certain degrees of autonomy and power, for example, daughters had equal inheritance rights to sons, and husbands could not sell property without their spouses’ consent^98^. In addition, widows were entitled to their dowry and to half of the couple’s assets^99^. The significance of women’s contributions to household finances, and their involvement in crafts and small urban enterprises, has also been studied, and although specific data on income, working environment, and affiliation to professional organizations are still limited, historical sources show women to be very active in milling and breadmaking and textile production^100,101^. Although a shorter breastfeeding period could be indicative of a higher fertility and population growth as well as an increasing involvement of women in economic activities, a reduced breastmilk supply should also be considered as being a possible cause for shorter breastfeeding patterns. Women who nurse their infants have historically been worried about low milk supply, seeking various methods such as diets featuring warm and moist foods (like chicken broths, egg yolks, dairy products, almonds and pine nuts), religious supplications through the intervention of saints, and the dedication of numerous sanctuaries. Instances of insufficient breast milk are not uncommon in historical sources and can be attributed to various factors, including inadequate nutrition^102^. No statistically significant difference in the isotopic values of women in Lisbon between the Roman, Early and Late Medieval period was observed, although for the Late Medieval period there is a higher intake of marine resources by all adults^67^, therefore the different pattern of breastfeeding might not be, in this case, connected to the poor maternal nutrition, but suggests that different variables can be at play such as cultural or religious practices, as well as societal and economic pressures.

Other sites dating to the Early Medieval period such as Setúbal, Beja, Torrão, and Silves, including individuals of Muslim faith, show a prolonged breastfeeding up to two or three years of age. In contrast, the Early Medieval Christian sites of Boadilla and Gozquez, nitrogen values in non-adults peak at around one year of age. Two Muslim individuals (BEJ1026 and BEJ3207) from Beja show a slightly earlier weaning pattern in dentine sequential analysis (Fig. 3) suggesting that variation in these practices certainly existed. In addition, it is important to take into account that variations among multiple tissues may be due to the bone turnover rate, or the physiological impact that stress could have had on young individuals, who did not survive into adulthood, compared to dentine obtained from adult individuals who lived longer^24^. The well-being of the nursing child was a central concern among Muslim religious scholars and physicians in the Medieval world. Breastfeeding was essential for the survival of the infants and breastmilk was considered to be very effective against any physical illness, as well as being able to pass physical and mental characteristics from the nursing individual to the child^103,104^. For these reasons animal milk was rejected and wet-nursing only considered in times of need. Interestingly, Islamic law also insisted that the minimal time for lactation should be two full years and cutting the breastfeeding shorter than twenty-one months was an act of injustice towards the infant^12^. This seem to be in agreement with all the Muslim sites included in this study with the exception of Early Medieval Lisbon^17^. A similar pattern between the Early and the Late Medieval period is also observed in Beja and Silves where non-adults δ^15^N values peak at age two during both periods. However, the introduction of a weaning food with relatively high δ^15^N values could also impact the curve, creating a more gradual decrease of the values. This is the case of the Late Medieval period individuals, aged up to four years old, at these sites still present enriched nitrogen values compared to the female baseline (ca. 1.5-2‰).

### Weaning practices and post weaning diets

The models created for this study with WARN allow for a detailed discussion of weaning practices including weaning timing, food sources, and the enrichment between the adult and juvenile isotopic values. The modelled weaning times of when complementary food was first introduced (t1) and termination of weaning (t2) present significant variations overall (SI Table 1). Sites can be grouped in three categories: Early introduction of solid food (within the first eight months) at the sites of Early Medieval Lisbon, Setúbal, Loulé, Boadilla and Gozquez; mid to late weaning initiation (twelve to eighteen months) in Roman Barcelona and Terrassa; late weaning initiation (over two years of age) at the sites of Roman Ibiza, and Early Medieval Torrão and Silves. This very late initiation of weaning seems unlikely as the breastmilk cannot provide alone the necessary nutrition for older infants, however, these values have also been reported elsewhere^105^ and seem to be influenced by the prior used in the WARN package that assumes an uncertainty of ± 3 years of age at both the onset (t1) and termination (t2) of weaning. The difficulty that the model is experiencing in detecting the start of the introduction of solid food into childhood diet at the sites of Roman Ibiza, Early Medieval Torrão and Silves might be due also to the type of weaning food that was chosen by these populations. These differences could be attributed to ecological diversity (for example, a focus on crops in inland regions) or cultural attitudes regarding the best timing for weaning. Great variability is also shown in the completion of weaning (ts2) (SI Table 1). Overall the majority of the populations complete weaning by the age of three (Roman and Early Medieval Lisbon, Early Medieval Setúbal, Beja, Boadilla and Gozquez and Late Medieval Lisbon). However some other sites, including Roman Terrassa, Early Medieval Loulé, Torrão, Silves, Late Medieval Silves shows the weaning concluded at age four. And one last group of sites including Roman Barcelona and Ibiza and Late Medieval Beja show completed weaning between the age of four and a half and five years old. The type of weaning food that was available to older children and what food sources were more likely to complement their diet should also be considered. A protein-dense food like aquatic resources, or the variation in diet of the breastfeeding woman could also have an impact in maintaining the δ^15^N values relatively high and therefore a clear change in the modelled curve at the end of the weaning might not be discernible. This pattern, however, has been observed before^106^ and could also highlight episodes of malnutrition and metabolic stress that can cause an increase in δ^15^N values^21,107^ in children, but it could also reflect a parental choice to reduce fertility with prolonged breastfeeding that would help in spacing out pregnancies. Alternatively, an increased reliance on breastfeeding due to a deficiency in suitable weaning foods or a general lack of food provision could also explain these isotopic values^108^.

Although the focus of this study was assessing weaning timing, the model also provides information about the estimated average δ^15^N value of complementary foods introduced to infants during weaning, and how those values differ from the δ^15^N values of the adult diet (SI Fig. 3). The Δ^15^N_adult−wnfood_ values are low (0.6‰ – 0.8‰) only for the sites of Boadilla and Late Medieval Silves, suggesting that at these sites weaning foods has a relatively close trophic level to the diet of the general population. The sites of Roman Lisbon, Ibiza and Terrassa and Early Medieval Setúbal show weaning food to be trophically higher than the previous populations (1.4‰ – 1.7‰) and the remaining Roman Barcelona, Early Medieval Lisbon, Beja, Loulé, Torrão, Silves, Gozquez, and Late Medieval Lisbon, Beja show even higher enrichments (2.2‰ – 3.9‰). For this last group, the inclusion of omnivore meat and aquatic resources is the most likely explanation as the Δ^15^N_adult−wnfood_ covers a trophic level. Interestingly the multi-period site of Lisbon exhibits a progressive rise over time when measuring Δ^15^N_adult−wnfood_ values. Notably, lower values were documented during the Roman period (1.0‰ – 2.3‰), while higher means occurred more frequently throughout Early Medieval times (1.8‰ – 2.9‰), with increased variability including even higher enrichments towards the Late Medieval period (0.9‰ – 3.5‰). The diet of the adult population in the Late Medieval period in Lisbon has been discussed elsewhere^67^, however, marine resources became preponderant in the adult population and it seems therefore likely that these would have been easily accessible as weaning and post weaning food.

## Conclusions

The results of this study have shown a great variety of breastfeeding and weaning patterns across time and space, suggesting that women’s adaptive and resilience strategies were at play in different socio-political milieus. Longer breastfeeding can foster longer pregnancy intervals, which in turn, result in better health and lower mortality rates for both mother and child^109^. In addition, both in the Roman and Muslim periods, breastfeeding practices were used to create bonds and kinship between children and families. In Roman society, sharing on the same breastmilk for two children from different mothers would provide a sibling status and it would be followed by patronage of that specific family^103^. In Islam breastfeeding is encouraged until the age of two, however, modern ethnographic studies have found that rural Berber women breastfeed for longer and milk kinship has also a long lasting tradition in both Muslim Berber and Arab cultures^104,110^. The latest findings in this study from multiple locations in Iberia present a combined depiction of the dietary habits during childhood and emphasize the role that women and children played in breastfeeding, weaning, and feeding customs. The results reveal an overall trend towards extended weaning periods among most of the explored dataset. However, it is yet to be verified whether this practice was a shared cultural norm or not. Although this study represents the most extensive examination on non-adult nutrition in this region to date, the authors hope that more data will be generated to allow for a deeper understanding of childrearing practices over several centuries in Iberia. To provide further insight into how the Iberian population adapted to changing socio-political conditions, additional analysis that incorporates incremental dentine isotope analysis with single compound amino acid isotope evaluation across evenly distributed age groups would be beneficial.

## Electronic supplementary material

Below is the link to the electronic supplementary material.

Supplementary Material 1

Supplementary Material 2

## Data Availability

Data is provided within the manuscript or supplementary information files.
