# Correction: Child-mother relationships and childhood dietary patterns in the Iberian Peninsula uncovered by Bayesian isotopic approaches

**DOI:** 10.1038/s41598-025-10454-8

**Published:** 2025-07-15

**Authors:** Alice Toso, Silvia Casimiro, Charlotte Oxborough, Simona Schifano, Maite I. García-Collado, Francisca Alves Cardoso, Joaquina Soares, Maria João Valente, Raquel Santos, Vanessa Filipe, Maria José da Silva Gonçalves, Nuno Neto, Paulo Rebelo, Rodrigo Banha da Silva, Anabela Novais de Castro Filipe, Michelle Alexander

**Affiliations:** 1 Bonn Center for ArchaeoSciences, Institut für Archäologie und Kulturanthropologie, Rheinische Friedrich-Wilhelms-Universität, Bonn, Germany; 2 IEM - Institute for Medieval Studies, School of Social Sciences and Humanities, Nova University of Lisbon, Portugal (NOVA FCSH) / LABOH CRIA – Center for Research in Anthropology (NOVA FCSH)/ Atalaia Plural - Archaeology, Heritage & Territory, Lisbon, Portugal; 3 Department of Archaeology. BioArCh, University of York, Environment Building, 2nd floor. Wentworth Way, YO10 5DD Heslington, UK; 4 Department of Geography, Prehistory and Archaeology. Research group in Medieval Archaeology, Heritage and Cultural Landscapes (IT442-22)., University of the Basque Country (UPV/EHU), Micaela Portilla Research Centre. 1 Justo Vélez de Elorriaga street., 01006 Vitoria-Gasteiz, Spain; 5 LABOH CRIA – Center for Research in Anthropology, School of Social Sciences and Humanities (NOVA FCSH), Nova University of Lisbon, Portugal/ In2PAST - Associated Laboratory for Research and Innovation in Heritage, Arts, Sustainability and Territory, Lisbon, Portugal; 6 Faculdade de Letras, UNIARQ - Centro de Arqueologia da Universidade de Lisboa, Universidade de Lisboa, Lisbon, Portugal; 7 Universidade do Algarve / CEAACP — Centro de Estudos de Arqueologia, Artes e Ciências do Património, Faro, Portugal; 8 Neoépica, Mem Martins, Portugal; 9 School of Social Sciences and Humanities (NOVA FCSH), Cota 80-86, IAP – Instituto de Arqueologia e Paleociências, Nova University of Lisbon, Lisbon, Portugal; 10 Museu de Arqueologia de Silves, Silves, Portugal; 11 CHAM – Center for Humanities, School of Social Sciences and Humanities, Nova University of Lisbon (CHAM - NOVA FCSH), Lisbon, Portugal; 12 Lisbon Archaeology Centre, (DMC/DPC/CAL), Lisbon City Council, Portugal; 13 Atalaia Plural, Lisbon, Portugal

Correction to: *Scientific Reports* 10.1038/s41598-025-97967-4, published online 13 April 2025

The original version of this Article contained errors.

In the Introduction, the objectives have been amended, where

“The objective of this paper is two-fold, addressing the following research questions:

1. What was the staple non-adult diet in the Iberian Peninsula and specifically southern Portugal over time?
2. Is a breastfeeding and weaning signal detectable in the population under consideration here and can a special weaning diet be identified among different socio-cultural and historical contexts?”

now reads:

“This study sets out to investigate two key aspects of early life dietary practices in southern Iberia. First, it aims to reconstruct the predominant dietary sources consumed by non-adult individuals across different temporal phases, with a particular focus on southern Portugal. Second, it explores whether isotopic signatures can elucidate patterns of infant feeding, notably the duration of breastfeeding and the nature of weaning foods, and how these may have varied in response to shifting historical and socio-cultural conditions.”

Furthermore, the references *Kragten 1994* and *Dunn and Carter 2018*, cited in the Materials and methods under the subheading ‘Collagen extraction and stable isotope analysis’, were omitted from the Reference list. The correct references are listed below.

Kragten, J. Calculating Standard Deviations and Confidence Intervals with a Universally Applicable Spreadsheet Technique. *The Analyst*, **119** (10), 2161–2166 (1994).

Dunn P. J. H. and J. F. Carter, eds., *Good practice guide for isotope ratio mass spectrometry*, 2nd Edition. FIRMS. ISBN 978-0-948926-33-4 (2018).

The references have been added to Reference list; subsequent references have been renumbered.

The original Article has been corrected.

