# Supplementary material for: Child-mother relationships and childhood dietary patterns in the Iberian Peninsula uncovered by Bayesian isotopic approaches
Source: Sci Rep. 2025 Apr 13;15:12704. doi: 10.1038/s41598-025-97967-4 (PMC11994822; doi:10.1038/s41598-025-97967-4)
Supplement: Supplementary file 1 — Supplementary Material 1 [file 41598_2025_97967_MOESM1_ESM.docx]

Supplementary information

# Archaeological context of the analysed sites

## Roman and Late Antique

The Roman and Late Antique burial grounds in this study are solely located in Lisbon. Most of the samples dating between the 2^nd^ and 4^th^ century CE, come from two sites related with the Northwest Necropolis of Olisipo - Praça da Figueira and Calçada do Lavra – distancing about 500m from each other. Both sites revealed the presence of the two most common funerary rituals at the time: inhumation and cremation ^1,2^. Information on these sites, burials and notable finds have already been detailed elsewhere ^1,3–11^, although both sites and collections continue to be studied due to its richness and complexity. The Lisbon dataset is complemented with samples from Rua Nova do Almada ^12^, and Largo do Conde Barão ^13^. Overall, these sites are representative of the Roman burial complexity. Both adults, male and females, and nonadult burials were recovered, alongside some secondary depositions mostly related to post-depositional disturbances.

The few samples from a later Late Antique burial grounds, dating from the 5^th^ to the 7^th^ century, were exhumed at Praça da Figueira ^4^, Rua da Prata ^14^ and Rua de São Nicolau ^15,16^. These later sites differ from the previous sites: for example, in these contexts, the graves of children aged between 6 months and 14 years old, were found without associated grave goods. Also, their small representation suggests that these were just small fragments of larger funerary spaces that have yet to be fully uncovered.

## Early medieval

Early medieval Muslim cemeteries included the sites of São Jorge Castle and Calçadinha do Tijolo, in Lisbon, dating between the 8th and 11th century. A full report of the excavation, dietary and anthropological study has been published elsewhere ^17,18^. These datasets were also incorporated into the present analysis.

In 2008/2009, an excavation was performed in Setúbal's Rua Francisco Flamengo as a rescue archaeology initiative. The site offers evidence of human presence from the Iron Age, but primarily revealed significant discoveries related to the Roman Imperial period and Islamic era. Some of the finds include Roman walls and pottery, accompanied by five Muslim burials dated between 980 and 1150 CE (Beta-256936:1000±40 BP) (Silva et al., 2010). In 2010, further investigations revealed fifteen Muslim burials present in this location. These graves were oriented southwest to northeast and positioned on the left side. Cross-sections also showed additional graves suggesting that the cemetery expanded beyond its intended limits ^19^. The discovery of these remains suggests an early use of the burial ground that was later abandoned after the Christian conquest during late medieval times.

A few kilometres east of Setúbal, a small rural Muslim cemetery was uncovered in Horta do Pinheiro. The site is characterised by burial that follows the Muslim rite of deposition of individuals on the right lateral side, with a southwest to northeast orientation ^20^, however no grave goods or settlement were found that could help to date the site more precisely and therefore the chronology is reported as 8th to 12th century ^21^.

Despite their divergent location (inland versus littoral/ coastline) Beja and Silves were two of the most important urban sites during the Early Medieval period in Portugal and were under Muslim rule between the 8^th^ and the 12^th^ century. An extensive cemetery was excavated in Beja and several smaller cemeteries were also recovered for the same period in Silves. An exhaustive description of the archaeological and dietary results for these sites is presented in ^22^.

The site of Prajo do Tejo (Quinta do Lago) is located in the modern-day natural park of Ria Formosa, 1600 meters from the current coastline, and about 9km south of Loulé. Archaeological research in the region provided evidence of dense human activity from the Roman period to the end of the Islamic rule (1^st^-12^th^/13^th^ centuries CE) and two main settlements have been uncovered in subsequent campaigns between 1984 and 2002 ^23^. An Islamic period settlement was excavated, including houses and a cemetery. The excavation uncovered five different domestic structures, two shell middens and two large rubbish pits. A number of materials dating to between the 11^th^ and 13^th^ century were recovered including a thimble and weaving tools ^23^. The cemetery included 76 individuals excavated in 1984 and 22 individuals recovered in 2001/2. The chronology is further confirmed by radiocarbon dating undertaken on two individuals dating respectively to 981-1160 cal AD (2σ Sac-1821) and 983-1159 cal AD (2 sigma; Sac-1892)^24^. Although the chronology fits with the one provided by the material culture, marine input into diet of these individuals - suggested by the amount of shells recovered at the site and its coastal location – might have affected their radiocarbon values producing an older chronology.

## Late medieval

Late medieval cemeteries have been excavated in Lisbon, Beja and Silves. The archaeological excavations conducted in Largo das Olarias and Quarteirão dos Lagares, located in the Mouraria neighbourhood of Lisbon, are likely parts of the same Islamic cemetery. Ongoing excavation efforts since 2015 have revealed a multitude of burial sites that suggests the identity of one of the primary Islamic cemeteries within Lisbon. Further research regarding site chronology, usage patterns and development history is ongoing, however, the complex stratigraphy of the site, and archaeological finds such as coins and pottery, suggest a late chronology for the main part of the cemetery (13^th^ – 15^th^ century). In addition, a small medieval Christian cemetery (14^th^-15^th^ century) has been uncovered in Poço do Borratem, just outside the Mouraria neighbourhood and was probably connected to the nearby hermitage of São Matheus. Previously published works provide a thorough description of the archaeological background for these sites as well as the zooarchaeological and dietary findings ^22,25,26^.

Late Medieval Christian burials were uncovered alongside Early Medieval burials in the same burial site in Beja. The chronology of this multi-faith cemetery was clarified through radiocarbon dating, which determined that these Christian burials date back to the 13th-15th century ^22,27^. In Silves, two separate Christian cemeteries were discovered and excavated: one situated on Rua Miguel Bombarda and another at Largo da Sé, both believed to originate from the same period (13th-15th century) ^28–30^. Similarly to the Early Medieval populations from these sites, the mean isotopic values for Late Medieval females ^22^ have been used as baseline to interpret and model the non-adult breastfeeding and weaning values for this study.


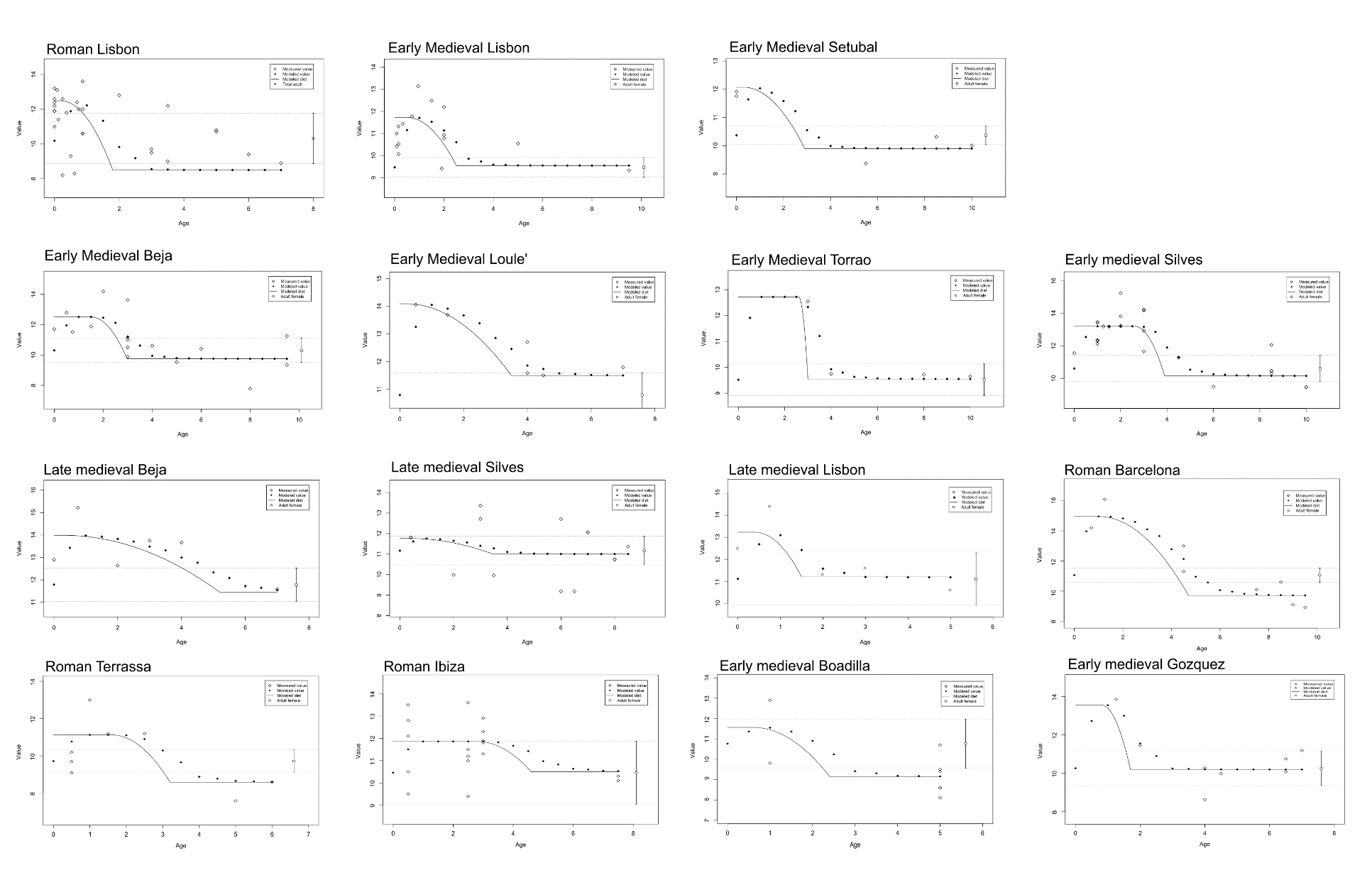


Fig. S1 δ^15^N_bone_ values with modelled bone and dietary δ^15^N values and adult female mean ± 1 SD


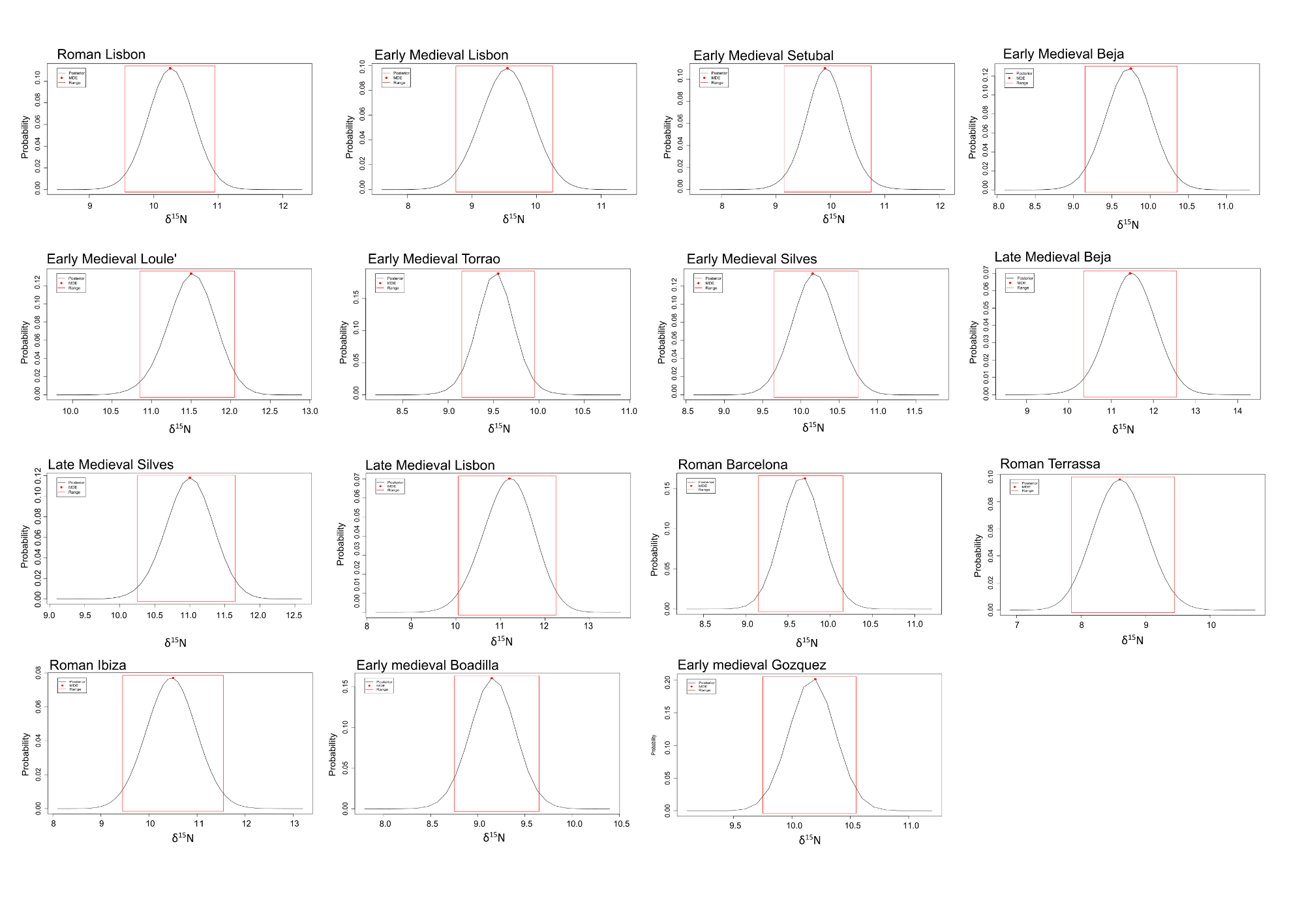
Fig. S2 δ^15^N_food_ values with modelled δ^15^N value of collagen synthesized entirely from weaning food.


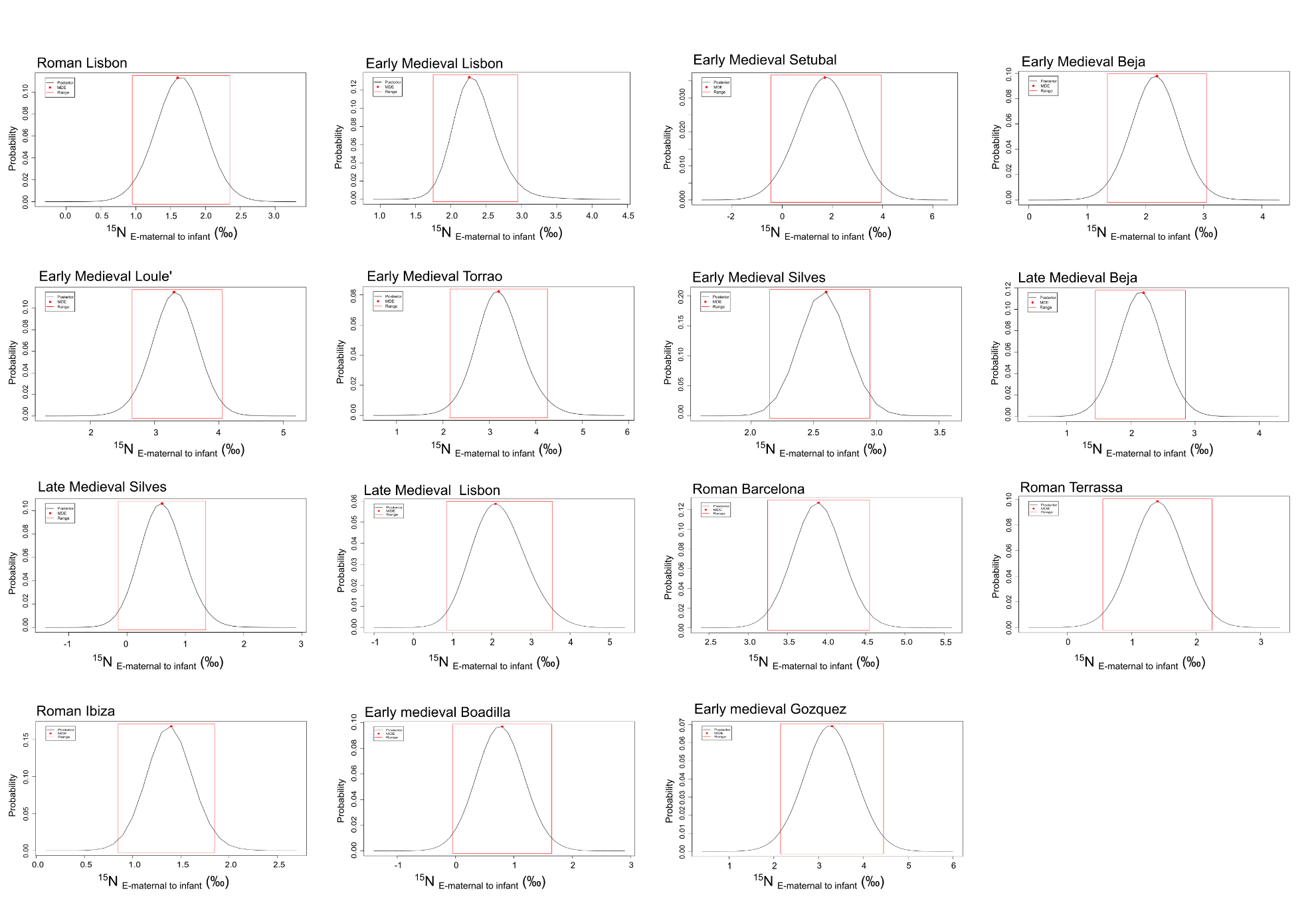
Fig. S3 δ^15^N_enrichment_ values with ^15^N-enrichment from maternal to infant tissue.


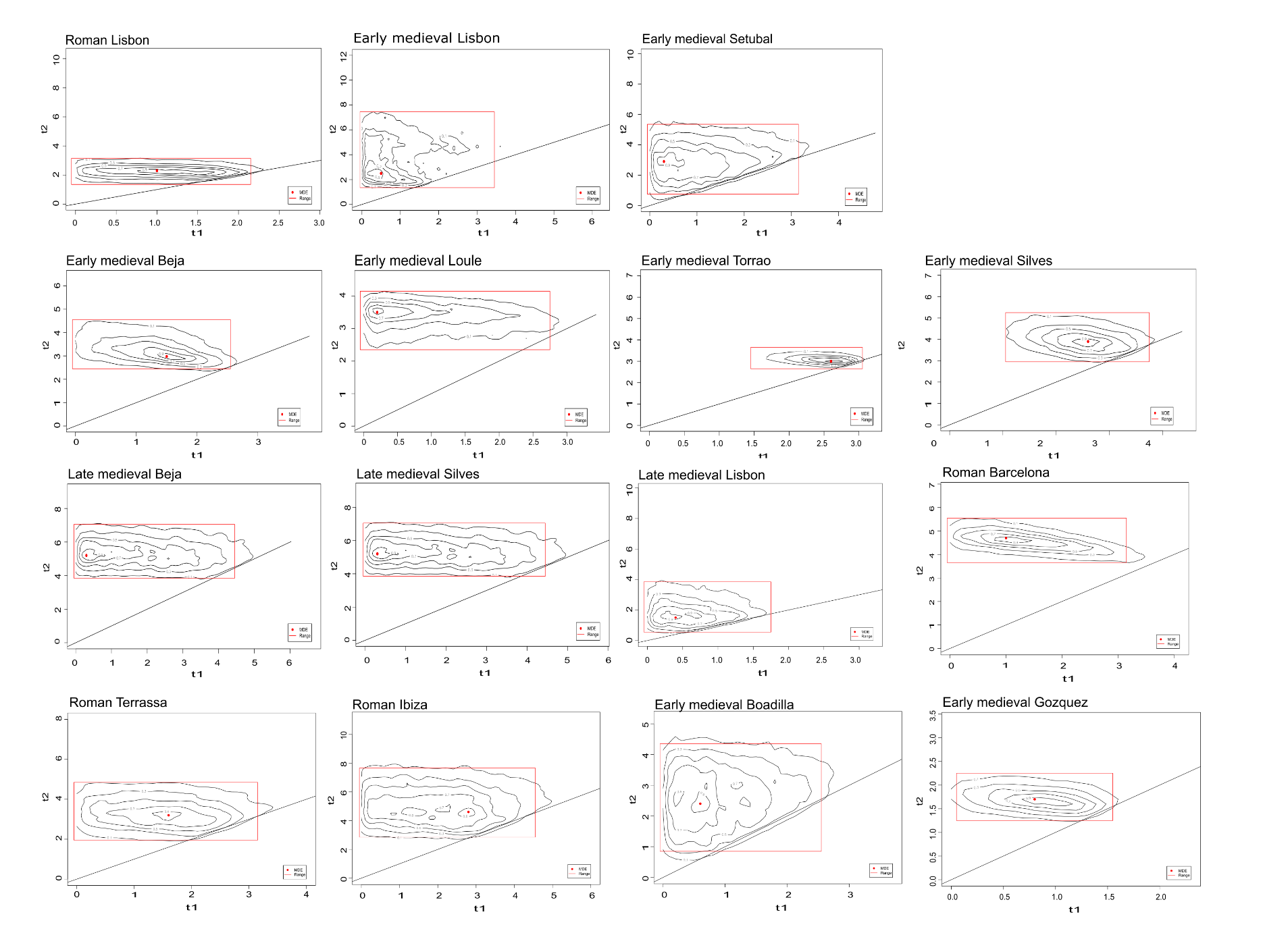
Fig. S4 Maximum density weaning ages plotted by the midpoint between onset (t1) and completion (t2) of weaning for each populations.


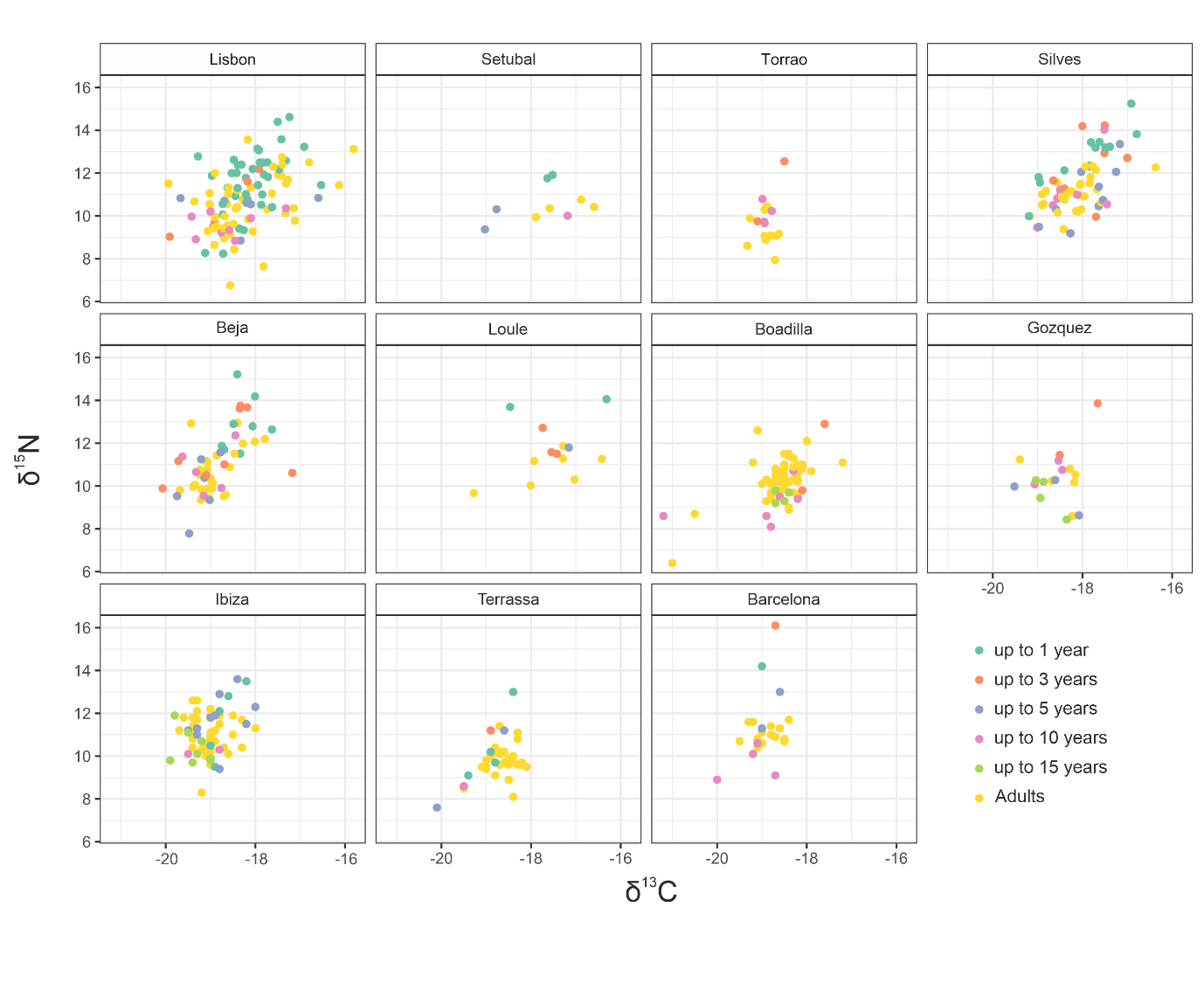


Fig.S5 δ^13^C and δ^15^N values for all the analysed populations divided by site.


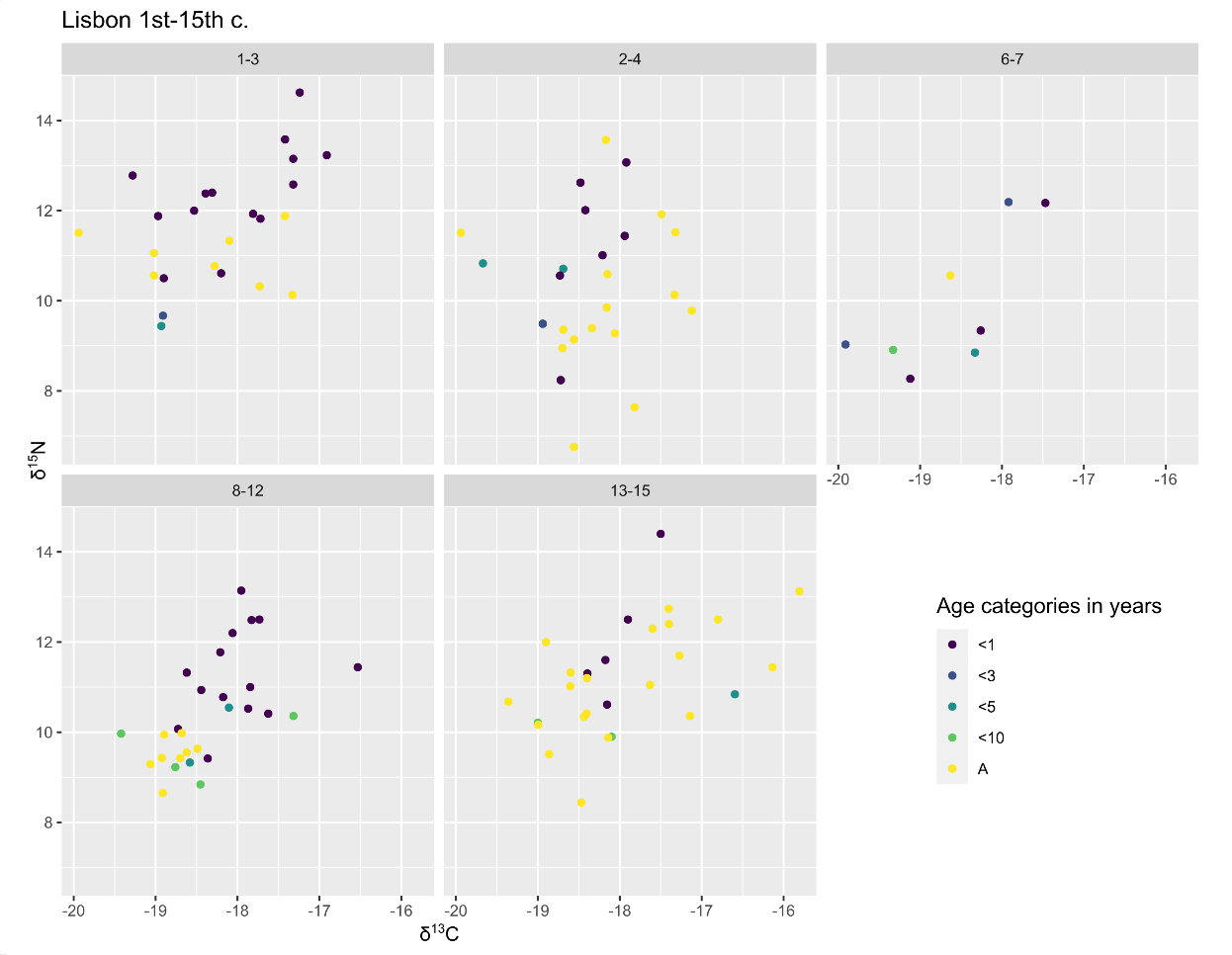


Fig.S6 δ^13^C and δ^15^N values for all the analysed populations in Lisbon divided by time period.

**Supplementary table title.**

Dataset SI (separate file).

Table S1. Summary statistics resulted from the Bayesian modelling (WARN R Package) for all human individuals with indication of Maximum density estimations (MDEs), Probability, Credible Intervals (CI), Adult female mean, Probability of Weaning start (t1) and completion (t2) and Mean squared distance.

Table S2. δ^13^C and δ^15^N values for the serial sections of dentine from six individuals from the Muslim and Christian burials in Beja.

**Table S3. δ^13^C and δ^15^N values for all human individuals, organized by site, faith, period (1-3= 1^st^-3^rd^ century; 2-4 = 2^nd^ – 3^rd^ century; 6-8 = 6^th^ - 8^th^ century; 8-12 = 8^th^-12^th^ century; 13-15 = 13^th^-15^th^ century ), sex (M=male; F=female, U= Undetermined sex) and age (A=adult; J= non-adult).**

1. Silva, R. B., Casimiro, S. & Alves-Cardoso, F. Praça da Figueira. in *Para além desta Vida: A memória funerária da cidade. Lisboa Romana - Felicitas Iulia Olisipo* (ed. Silva, R. B.) 44–65 (Câmara Municipal de Lisboa, Lisbon, 2021).

2. Rebelo, P., Peça, P., Rocha, M. & Bolila, C. *Relatório Final: Intervenção Arqueológica Na Calçada Do Lavra, N^o^2 a 10, Lisboa*. (2020).

3. Casimiro, S., Silva, R.B., Alves Cardoso, F. ‘Et Sepultus Est’: A Multiplicidade da Morte na Necrópole Noroeste de Olisipo. *Arqueologia & História* **71–72**, 25–33 (2022).

4. Casimiro, S., Antunes Ferreira, N., Alves Cardoso, F. A infância entre a Época Romana e a Antiguidade Tardia. in *Para além desta Vida: A memória funerária da cidade. Lisboa Romana - Felicitas Iulia Olisipo* (ed. Silva, R. B.) 174–181 (Câmara Municipal de Lisboa, Lisbon, 2021).

5. Casimiro, S., Alves Cardoso, F., Silva, R.B., Assis, S. Requiscat in Pace - Abordagem transdisciplinar a possíveis casos de enterramentos atípicos identificados na Necrópole Noroeste de Olisipo. in *Arqueologia em Portugal - O Estado da Questão* (ed. Arnaud J Martins) 1215–1227 (Associação dos Arqueólogos Portugueses, Lisbon, 2017).

6. Alves Cardoso, F., Casimiro S., Garcia, S., Antunes Ferreira, N., Granja, R., Lourenço, M., Duarte, C., Gonçalves, D. Os Olisiponenses: Estudo bioantropológico de uma população da Lusitania. in *Para além desta Vida: A memória funerária da cidade. Lisboa Romana - Felicitas Iulia Olisipo* (ed. Silva, R. B.) 161–173 (Câmara Municipal de Lisboa, Lisbon, 2021).

7. Peça, P., Bolila, C., Granja, R., Rebelo, P. Calçada do Lavra: Testemunho da variabilidade de rituais funerários em época romana. in *Para além desta Vida: A memória funerária da cidade. Lisboa Romana - Felicitas Iulia Olisipo* (ed. Silva, R. B.) 75–83 (Câmara Municipal de Lisboa, Lisbon, 2021).

8. Morrone, A. The Buried cupae of Praça da Figueira, Lisbon. Contributions to the scientific study of archaeological cremations. (Cranfield University, 2017).

9. Vieira, V. As lucernas Romanas da Praça da Figueira (Lisboa): Contributo para o conhecimento de Olisipo. (Universidade Nova de Lisboa, 2011).

10. Silva, R. B. As marcas de oleiro em terra sigillata da Praça da Figueira: uma contribuição para o estudo da economia de Olisipo (Séc.I a.C.- Séc.II d.C.). (2005).

11. Silva, R. B. As marcas de oleiro na terra sigillata e a circulação de vasos na Península de Lisboa. (Universidade Nova de Lisboa, 2012).

12. Pinheiro, H., Granja, R., Neto, N. Rua Nova do Almadan^o^63-73: Vestigios da necrópole romana da “via Este-Oeste” de Olisipo. in *Para além desta Vida: A memória funerária da cidade. Lisboa Romana - Felicitas Iulia Olisipo* (ed. Silva, R. B.) 40–43 (Câmara Municipal de Lisboa, Lisbon, 2021).

13. Neto, N., Rebelo, P. & Brito, S. *Relatório Final: TIntervenção Arqueológica No Palacio Conde Barão de Alvito*. (2020).

14. Casimiro, S., Manso, C., Neto, N., Reis, J., Oliveira, J.M., Alves Cardoso, F. Rua da Prata: Evidências fúnebres da Antiguidade Tardia. in *Para além desta Vida: A memória funerária da cidade. Lisboa Romana - Felicitas Iulia Olisipo* (ed. Silva, R. B.) 121 (Câmara Municipal de Lisboa, 2021).

15. Casimiro, S., Oliveira, J.M., Manso, C., Silva, R.B., Seabra, A. Rua de São Nicolau e Corpus Christi: Discretas evidências da Antiguidade Tardia. in *Para além desta Vida: A memória funerária da cidade. Lisboa Romana - Felicitas Iulia Olisipo* (ed. Silva, R. B.) 120 (Câmara Municipal de Lisboa, 2021).

16. Casimiro, S. & Silva, R. B. Enterramentos infantis na Rua de São Nicolau (Lisboa). in *Arqueologia em Portugal - 150 anos* (ed. Arnaud, J., Martins, A., Neves, C.) 859–863 (Associação dos Arqueólogos Portugueses, Lisbon, 2013).

17. Toso, A., Gaspar, S., Banha da Silva, R., Garcia, S. J. & Alexander, M. High status diet and health in Medieval Lisbon: a combined isotopic and osteological analysis of the Islamic population from São Jorge Castle, Portugal. *Archaeol. Anthropol. Sci.* (2019) doi:10.1007/s12520-019-00822-7.

18. Filipe, V., Toso, A., Inocêncio, J. Perspectivas arqueobiológicas sobre a necrópole islâmica de Alfama. in *I Encontro de Arqueologia de Lisboa: Uma Cidade em Escavação* (CAL/DPC/DMC/CML, Lisboa, 2018).

19. Silva, C. T. da, Soares, J., Coelho-Soares, A., Duarte, S. & Godinho, R. Preexistencias de Setúbal. 2^a^ campanha de escavações arqueológicas na Rua Francisco Augusto Flamengo, nos 10-12 Da Idade do Ferro ao Período Medieval. *Musa* **4**, 161–214 (2014).

20. Boone, J. L. & Benco, N. L. Islamic Settlement in North Africa and the Iberian Peninsula. *Annu. Rev. Anthropol.* **28**, 51–71 (1999).

21. Mathias, H., Simão, I. & Nunes, T. *Minimização de Impactes Sobre o Património Cultural Decorrentes Da Execução Dos Blocos de Rega de Vale Do Gaio (Fase de Obra) e Do Adutor de Vale Do Gaio (Troço 4): Fase de Obra. Sondagens Arqueológicas. Horta Do Pinheiro 5*. (2016).

22. Toso, A. *et al.* Beyond faith: Biomolecular evidence for changing urban economies in multi-faith medieval Portugal. *Am. J. Phys. Anthropol.* (2021) doi:10.1002/ajpa.24343.

23. Arruda, A. M., Almeida, R. R. & Freitas, V. O sítio islâmico do Tejo do Praio, Quinta do Lago, Loulé: uma primeira análise e caracterização. *Xelb* **4**, 247–264 (2004).

24. Vilhena de Carvalho, M. L., Ferreira, M. T., Silva, A. M. & Cunha, E. Lesiones traumáticas en la populación islámica de Tejo do Praio (Portugal). in *Actas del VII Congresso Nacional de Paleopatología.* 428–432 (Universidad de Las Islas Baleares, 2005).

25. Filipe, V., Vieria, V., Ferro, S., Pereira, T. & Neves, D. *Relatório Final Dos Trabalhos Arqueológicos Desenvolvidos No Poço Do Borratém No Âmbito Do Projeto de Instalação de Ecopontos Subterrâneos Na Cidade de Lisboa. Relatório Policopiado Direção Geral Do Património Cultural*. (2020).

26. Valente, M. J. & Marques, A. Alimentação mudéjar em Lisboa: dados preliminares sobre a zooarqueologia do Largo da Severa (Mouraria, Lisboa). in *Diz-me o que comes... alimentação antes e depois da cidade* (eds. Senna Martinez, J. C. et al.) vol. 1 (ACDPRINT, S.A, Lisboa, 2017).

27. Gomes, M., Santos, R. & Vilhena de Carvalho, M. L. *Intervençãao Arqueológica Na Escola Secundária Diogo Gouveia – Beja*. (2014).

28. Casimiro, T., Chanoca, C. & Vieira, A. I. Silves Polis: duas necrópoles medievais cristãs. *Xelb* **8**, (2008).

29. Gamito, T. J. A cisterna árabe da Rua do Castelo – Silves. *Xelb* **4**, 235–246 (2003).

30. Gamito, T. J., Lensch, G., Marschall, K., Oliveira, L. & Veiga, I. A. da. A Sé de Silves: a memória da pedra. *Arqueologia Medieval* **5**, 227–293 (1997).
